# Supporting Breastfeeding in the Setting of Perinatal Opioid Use Disorder

**DOI:** 10.1111/jmwh.70122

**Published:** 2026-04-09

**Authors:** Lindsey A. Baksh, Stephanie M. Hartwig

**Affiliations:** ^1^ Vanderbilt University School of Nursing Nashville Tennessee; ^2^ Department of Obstetrics and Gynecology Vanderbilt University Medical Center Nashville Tennessee

**Keywords:** Breastfeeding, neonatal opioid withdrawal syndrome, opioid use disorder, pregnancy, postpartum

## Abstract

Studies have shown that many pregnant women with opioid use disorder intend to initiate breastfeeding after giving birth. However, continuation rates beyond the initial postpartum hospitalization are low. Achieving and maintaining an antepartum breastfeeding plan may be complicated by the unique challenges that opioid‐exposed dyads experience after birth. Stigma, inconsistent messaging about the safety of breastfeeding while on opioid agonist therapy, and institutional policies that separate the dyad can contribute to low continuation rates. Additionally, neonatal opioid withdrawal syndrome may include gastrointestinal, musculoskeletal, and neurological symptoms that can lead to difficulty establishing breastfeeding. Neonatal weight loss may require temporary supplemental formula use. The safety and benefits of breastfeeding in the setting of opioid agonist treatment for perinatal opioid use disorder is well established in the literature. This case reviews the safety and benefits of breastfeeding in the setting of perinatal opioid use disorder and provides evidence‐based strategies to support the feeding preferences of these families.

## CASE SUMMARY

*J.S. is a 31‐year‐old primigravid person who presented at 12 weeks’ estimated gestational age for a new prenatal visit. She reported that she had opioid use disorder (OUD) and was currently enrolled in a local opioid treatment program. Her treatment plan included 12 mg of buprenorphine/naloxone (Suboxone) sublingually daily, weekly group therapy, and recovery support from a peer support specialist*.

*J.S. was accompanied by her partner. This pregnancy was unexpected, but they were excited about starting a family. J.S. said her opioid treatment program recommended a specific midwifery practice due to the supportive care patients with OUD received. J.S. told her midwife she had questions about the safety of buprenorphine during pregnancy and breastfeeding. J.S. had heard from friends that “you can't breastfeed on Suboxone” and that taking buprenorphine/naloxone meant her newborn* *will have “really bad withdrawal and will be in the hospital for a month.”*

*In addition to buprenorphine/naloxone 12 mg sublingually daily, J.S. was also taking prenatal vitamins. She reported no alcohol or tobacco use. J.S. also informed the midwife that she was treated for hepatitis C (HCV) last year and was worried that this would prevent her from breastfeeding. J.S. provided consent for a urine drug screen and blood testing for the HCV antibody and HCV RNA. The urine drug screen was positive for buprenorphine, and the HCV antibody test was positive with a negative viral load, indicating prior treatment and no active infection*.

*J.S. continued her prenatal care with the midwife. At 32 weeks, J.S. reported an increase in opioid cravings and informed the midwife that the opioid treatment program increased her buprenorphine/naloxone dosage to 16 mg sublingually daily. She reported that her cravings reduced, and she continued to abstain from opioids*.

*J.S. reported that her opioid treatment program recommended a third trimester lactation consultation. The midwife made this referral, and J.S. met with a certified lactation consultant at 37 weeks. The lactation consultant provided written and verbal education about neonatal opioid withdrawal syndrome (NOWS), including the hospital policy to observe newborns exposed to opioids in the hospital for 4 to 7 days. J.S. also received information about the maternal and newborn benefits of providing breastmilk during this time. J.S. was provided reassurance that she could provide breastmilk since her testing indicated she was no longer infected with HCV. A breast pump was ordered, and follow‐up information was provided as needed*.

*At 38 weeks, J.S. presented to the labor and delivery unit in active labor; her buprenorphine/naloxone dosage was still 16 mg daily, and she had been abstinent from opioids for more than 30 days. Labor progress was uncomplicated, and J.S. chose epidural analgesia for comfort during labor. J.S. gave birth vaginally to a healthy, vigorous male newborn who weighed 7 lbs 11 oz with an Apgar score of 7 and 9 at 1 and 5 minutes. J.S.’s newborn was diagnosed with NOWS on day of life 3. A family room was provided for J.S. to room‐in with her newborn. Despite the diagnosis of NOWS, withdrawal symptoms remained mild and decreased with nonpharmacologic measures. During this time, J.S. was able to breastfeed, provide a low‐stimulation environment, and learn techniques to console her newborn. The pediatric providers determined that pharmacologic treatment was not indicated, and her newborn was eligible for discharge on day of life 6*.

*At her 2‐week postpartum follow‐up visit, J.S. told the midwife breastfeeding was going well, but her mother told her that breastfeeding “isn't safe” and suggested she use formula instead. The midwife reassured J.S. that breastfeeding was compatible with buprenorphine/naloxone and reinforced availability of pediatric providers, lactation consultants, and online resources such as HealthyChildren.org and MotherToBaby. At the 6‐week postpartum visit, J.S. was exclusively breastfeeding, attending regular support group meetings, and successfully maintaining opioid abstinence on 16 mg of buprenorphine/naloxone daily*.

*Note that this case summary was a composite of elements from different patients*.

## INTRODUCTION

Approximately 5% of pregnant individuals use at least one addictive substance, most commonly alcohol, tobacco, and cannabis.^1^ Opioid use in pregnancy has also become more common due to increasing rates of opioid use among adults in the United States. Between 2010 and 2017, the number of pregnancies complicated by opioid use disorder (OUD) increased dramatically from 3.5 to 8.2 per 1000 births.^2^ In utero opioid exposure, including exposure to medications for OUD (MOUD), can result in neonatal opioid withdrawal syndrome (NOWS). Rates of NOWS in the United States have increased from 4 to 7.3 cases per 1000 births between 2010 and 2017.^2^ After birth, neonatal care prioritizes nonpharmacologic interventions such as breastfeeding, rooming‐in, and skin‐to‐skin contact.^3^ Breastfeeding is recommended by professional organizations for infants exposed to maternal MOUD such as methadone, buprenorphine, and buprenorphine/naloxone.^4^, ^5^, ^6^ Despite these recommendations, however, rates of breastfeeding continuation beyond the birth hospitalization are low.^7^, ^8^ Medication exposure, inaccurate information from health care providers, and fears related to hepatitis C (HCV) infection have been reported as barriers to breastfeeding among women on MOUD. Midwives and other health providers are uniquely positioned to support patients with OUD in creating and implementing a sustained feeding plan that includes provision of breastmilk.

### Perinatal OUD

Although rates vary by state, the rate of perinatal OUD at the time of labor and birth hospitalization is approximately 8.2 per 1000 births.^2^ Any prenatal opioid exposure increases the risk of health consequences for the birthing person and neonate. Opioid use is associated with adverse health outcomes including overdose, infection, severe maternal morbidity, and death.^4^ Overdose is a leading cause of mortality in the United States, and in 2020, 25% of pregnancy‐related deaths were attributed to substance use disorders.^9^, ^10^ Fetal and neonatal risks of in utero opioid exposure include preterm birth, low birth weight, and NOWS.^6^ Pregnant individuals with untreated OUD are less likely to attend prenatal care visits, often due to lack of insurance coverage, transportation, or childcare. Attendance at prenatal care visits can reduce the risk of adverse outcomes for the dyad.^11^

### Treatment of Perinatal OUD

Treatment of perinatal OUD significantly improves outcomes for the birthing person and neonate and includes the use of medications such as methadone, buprenorphine (Subutex), buprenorphine/naloxone (Suboxone), naltrexone and abstinence‐based therapies (Table 1).^11^ These medications are associated with reduced risk of adverse maternal and neonatal outcomes and are safe for use during pregnancy and breastfeeding.^4^, ^5^ Methadone and buprenorphine, including buprenorphine/naloxone, are considered the gold standard of treatment for perinatal OUD.^4^, ^9^ Historically, sublingual buprenorphine monoproduct was used during pregnancy and lactation due to concerns about naloxone exposure.^4^, ^12^ Recent research has demonstrated not only the safety of buprenorphine/naloxone, but also indicated improved neonatal outcomes compared with buprenorphine monoproduct.^12^ Monthly extended‐release buprenorphine preparations have limited safety data in pregnancy and lactation.^13^

**Table 1:** Medications for Opioid Use Disorder

Medication Name	Brand Name(s)	Mechanism of Action	RID, %	Breastfeeding Recommendations	Clinical Considerations
Buprenorphine	Subutex, Brixadi, Sublocade	Partial agonist at mu‐opioid receptors^13^	0.1‐2.5	Compatible; no dosage limitation^5^ Limited safety data on extended‐release formulations^13^	Available in sublingual and extended‐release formulations^4^ Higher abuse potential^4^
Buprenorphine/naloxone	Suboxone	Partial agonist at mu‐opioid receptor combined with opioid antagonist^13^	N/A	Compatible; no dosage limitation^5^	Sublingual film strip or tablet^13^ Naloxone component added to deter misuse but is not bioavailable with sublingual administration^4^
Methadone	Methadose, Dolophine	Full opioid agonist^13^	1‐3	Compatible; no dosage limitation^5^ Monitor for sedation if dose is greater than 100 mg^24^	Must be dispensed by a federally licensed opioid treatment program^4^ Requires daily in‐person dosing^4^ Dose increases in the third trimester are common^4^
Naltrexone	Vivitrol	Opioid antagonist^5^	0.83 ‐1	Compatible; limited safety data^5^	Available in monthly injection or daily oral tablet^4^, ^5^

Abbreviations: N/A, not available; RID, relative infant dosage.

Compared to no treatment, treatment with buprenorphine products or methadone result in reduced rates of preterm birth, stillbirth, low birth weight, and neonatal intensive care unit admission.^9^ Compared to methadone, treatment with buprenorphine products are associated with less severe withdrawal symptoms, lower rates of NOWS, and shorter newborn length of stay.^14^ Initiation of MOUD early in pregnancy and higher therapeutic doses of buprenorphine products have been associated with increased treatment retention.^15^ Thus, physiologic changes to plasma volume and drug metabolism in the third trimester often result in increased MOUD doses.^4^

Although less commonly utilized, other treatment options for perinatal OUD include naltrexone and abstinence‐based treatment. Long‐term outcome data on naltrexone exposure in pregnancy and lactation are lacking; however, it is not contraindicated.^4^, ^5^ Abstinence‐based treatment is not preferred but may be offered to patients who lack access or prefer to avoid MOUD.^4^ Abstinence‐based treatment carries an increased risk of relapse and overdose compared with buprenorphine and methadone. Treatment of perinatal OUD should be customized based on availability and the woman's preference.^4^, ^15^

### Neonatal Opioid Withdrawal Syndrome

After birth, neonates with chronic in utero exposure to any opioid experience an abrupt discontinuation that results in a cluster of symptoms called NOWS. Withdrawal symptoms are an expected and treatable side effect of in utero opioid exposure.^3^, ^6^ Although maternal treatment with MOUD improves outcomes, the risk of NOWS is not eliminated.^3^, ^6^, ^14^ The presence and severity of withdrawal symptoms is unpredictable. For neonates exposed to methadone or buprenorphine products, the risk is unrelated to the maternal dose of medication.^9^, ^14^ Thus, newborns exposed to opioids are typically kept in the hospital for 4 to7 days and monitored for withdrawal symptoms using a standardized screening tool such as the Finnegan Neonatal Abstinence Scoring System or the Eat, Sleep, and Console approach.^3^, ^6^

Opioid withdrawal in the newborn primarily affects the central nervous system and the gastrointestinal tract.^3^, ^6^ Clinical symptoms may appear within 24 hours or be delayed until day of life 5 to 7.^3^, ^6^ A full‐term newborn exposed to opioids may exhibit increased muscle tone, irritability, high‐pitched crying, difficulty feeding, loose stools, and excessive weight loss.^3^, ^6^ Strategies that prioritize keeping the dyad together promote bonding and facilitate breastfeeding.^3^, ^14^ Other nonpharmacologic interventions such as rooming‐in, providing a low‐stimulation environment, swaddling, cluster care, and skin‐to‐skin time are first‐line interventions for NOWS.^3^, ^6^, ^14^ Severe NOWS is treated with pharmacologic agents such as morphine, methadone, or buprenorphine.^3^, ^6^, ^14^

## BREASTFEEDING AND PERINATAL OUD

### Benefits and Barriers

There are specific breastfeeding benefits for newborns exposed to opioids. Breastfeeding promotes maternal‐infant bonding, can reduce the severity of NOWS, and can shorten the neonatal length of stay.^3^, ^14^ Improved maternal‐infant bonding may reduce stress and subsequently support breastfeeding and attendance at postpartum visits.^5^, ^16^, ^17^ Breastfeeding has been associated with increased OUD treatment retention, which is in turn associated with a reduced risk of maternal overdose.^15^, ^18^ Decreasing the newborn length of stay results in cost‐savings.^3^, ^6^

Qualitative studies have shown that many pregnant individuals treated with MOUD intend to breastfeed and initiate breastfeeding; however, rates vary between 8% to 81%.^17^ In 2023, the PRAMS survey found 83% of women who self‐reported antepartum opioid use attempted breastfeeding.^8^ Despite high levels of breastfeeding intention and initiation, individuals who reported opioid exposure antenatally had a statistically significant shorter breastfeeding duration and were less likely to breastfeed at 6, 10, and 20 weeks postpartum compared with respondents who did not report opioid exposure.^8^

Standish et al interviewed 23 individuals between 1 to 7 months postpartum who attended specialty clinics for women with OUD and their infants.^19^ Among the 70% of participants who initiated breastfeeding, 25% reported breastfeeding less than 1 week, 25% between 1 to 4 weeks, 25% between 5 to 8 weeks, and 25% greater than 8 weeks. No women reported having postpartum lactation support, despite ongoing breastfeeding challenges. Stephen et al found that only 23% of women with OUD were still providing breast milk at 6 months postpartum, compared with approximately 50% of those without OUD.^7^ Multidisciplinary perinatal‐addiction clinics that can provide buprenorphine have improved breastfeeding initiation and continuation.^20^

Postpartum individuals with OUD often face challenges to successful breastfeeding, leading to early discontinuation.^7^ Pregnant people with OUD report receiving general education about the benefits of breastfeeding; however, information specific to breastfeeding a newborn exposed to opioids varies.^16^, ^19^, ^20^, ^21^ Fear of medication transfer into breastmilk has been reported as a barrier to breastfeeding.^22^ Medications with a relative infant dosage of less than 10% are generally considered safe during lactation.^5^ Buprenorphine products and methadone are excreted in breastmilk at low concentrations, with relative infant doses of 0.38% and 3%, respectively.^5^, ^13^, ^23^ The maternal dosage of buprenorphine or methadone is not associated with infant plasma or urine concentrations.^5^, ^13^, ^23^, ^24^ Additionally, buprenorphine products have poor oral bioavailability, which also contributes to reduced infant exposure. Adverse effects of MOUD exposure via breastmilk are rare; however, drowsiness and poor feeding have been reported.^5^, ^13^, ^23^, ^24^

Full‐term infants exposed to opioids may also experience excessive weight loss from NOWS symptoms and require formula supplementation. Because infant weight loss may be interpreted as poor milk production, there may be a decrease in breast milk production if the lactating person is not supported in maintaining milk supply during formula supplementation.^5^ Clinicians may be unaware of breastfeeding recommendations, resulting in patients receiving conflicting information.^17^ Unexpected difficulty with latch or weight loss can be discouraging and result in early discontinuation of breastfeeding.^16^, ^17^, ^22^, ^25^

Preterm birth or newborns with additional medical complications may further complicate breastfeeding initiation. Provider, family, and partner support or lack of support can also impact breastfeeding. Individuals with OUD who report increased support are more likely to breastfeed.^16^, ^17^ Additional concerns that may impact the decision to continue breastfeeding include uncertainty regarding the safety of MOUD transmission through breastmilk, experiencing stigma from health care providers after birth, and competing demands related to treatment responsibilities.^16^, ^17^, ^22^, ^25^

For individuals on MOUD who are not actively using illicit substances, breastfeeding is recommended by the American College of Obstetricians and Gynecologists, the American Academy of Pediatrics, and the Academy of Breastfeeding Medicine.^4^, ^5^, ^6^ Women who return to active illicit substance use should be advised to discontinue breastfeeding or pump and discard breastmilk until substances are safely cleared. Risks associated with breastfeeding during active substance use include reduced parental ability to respond to infant feeding cues as well as infant substance exposure through breastmilk. Infant exposure carries a risk of acute toxicity, reduced breastfeeding ability, and possible changes in neonatal brain development.^5^

## CLINICAL PRACTICE IMPLICATIONS

### Antepartum Education

Midwives and clinicians providing prenatal care to individuals with OUD should discuss feeding preferences throughout the antepartum period. In the absence of anticipatory guidance about NOWS and navigating breastfeeding challenges, patients express surprise and confusion that breastfeeding can be more difficult with a newborn exposed to opioids.^19^ During the third trimester, clinicians should provide education about the benefits and safety of breastfeeding while taking MOUD. Anticipatory guidance should include neonatal symptoms, management strategies, and institution‐specific policies that may prolong neonatal hospitalization.^21^, ^25^ Health care providers caring for patients with OUD should discuss potential limiting factors for breastfeeding to set reasonable expectations. Date and type of last illicit substance use, concomitant medications, and HIV status can all impact breastfeeding to varying degrees.^5^, ^6^, ^21^

### Support Immediately Postpartum

Illicit substance use around the time of birth can influence whether breastmilk can be provided immediately or if there is a need to pump and discard breastmilk until substances are cleared. Institution‐specific policies regarding breastfeeding after relapse vary and may prevent breastfeeding initiation. In the setting of active HCV (positive RNA viral load), breastfeeding is safe if the woman's nipples are not cracked or bleeding.^4^ If cracked or bleeding nipples occur, milk should be expressed and discarded until cracks are healed and no longer bleeding.^4^, ^6^

Infants exposed to opioids may need inpatient observation beyond the routine postpartum hospitalization. Separation of the dyad during this time is a barrier to breastfeeding.^17^ The health care team can support breastfeeding by advocating for rooming‐in and providing ongoing lactation support, including an electric breast pump.^16^, ^17^ Education should also include the symptoms of NOWS, how to console the newborn, potential feeding challenges, and nutritional supplementation options.^16^, ^17^

Infants with NOWS may have neurological or gastrointestinal symptoms that make breastfeeding more challenging, which may result in rapid weight loss.^6^ Weight loss may require formula supplementation or recommendations to cease breastfeeding altogether.^3^, ^16^, ^17^, ^19^ If supplementation is required, health care providers can offer information about how to express and maintain milk supply. Birthing people have reported that breastfeeding difficulties often start during the birth hospitalization, and the loss of support upon discharge may result in early cessation.^16^, ^22^

### Ongoing Support

Long‐term breastfeeding support for birthing people with OUD includes continued postpartum substance use treatment and continuation of MOUD.^15^, ^26^ Stressors during the postpartum period increase the risk of return to illicit substance use, overdose, and death. Treatment for OUD should be continued during the postpartum period because it is associated with increased treatment engagement and reduced risk of overdose.^11^, ^18^, ^26^ Access to efficacious interventions for OUD vary based on several factors, including geography, race, ethnicity, and stigma, and can greatly affect treatment trajectories.^7^

Research suggests that breastfeeding may be a protective factor against OUD treatment dropout.^15^, ^18^ However, breastfeeding is not recommended in the setting of active illicit substance use. Individuals who experience a relapse and want to continue providing breastmilk should pump and discard milk until the substance is cleared. The Academy of Breastfeeding Medicine Clinical Protocol #21 (2023) provides information about specific substances and their clearance timeline.^5^

### Advocacy

Health care providers can support families impacted by OUD by advocating for rooming‐in, developing evidence‐based protocols regarding breastfeeding the newborn exposed to opioids, and care coordination with addiction treatment providers. Insurance providers, including state Medicaid plans, cover the cost of a breast pump, but patients may need assistance navigating the process. The United States does not require employers to provide paid parental leave, and birthing people with OUD have reported discontinuing breastfeeding upon their return to work.^17^ Clinicians can support long‐term breastfeeding by providing information regarding breast milk expression and milk storage. Collaborative relationships between women's health providers and opioid treatment programs can facilitate timely entry into prenatal care and ensure pregnant individuals receive evidence‐based information and support for their feeding preferences. If formal collaborative relationships are not available, knowledge about local treatment programs will help pregnant and postpartum individuals access OUD treatment during and after pregnancy.

## CONCLUSION

In the case summary, J.S. received supportive, evidence‐based care from her midwife, including support to continue buprenorphine/naloxone during pregnancy and breastfeeding. A collaborative relationship existed between the opioid treatment program and the midwifery practice, which facilitated J.S. receiving timely and supportive antepartum care. J.S. received education during the third trimester that focused on the benefits of breastfeeding in the setting of OUD as well as NOWS education. During the immediate postpartum period, the dyad was able to breastfeed, room‐in, and prioritize nonpharmacologic interventions to reduce NOWS symptoms. Further, J.S. received a breast pump and support from a lactation consultant before and after hospital discharge. The midwife in this case provided ongoing postpartum lactation support, evidence‐based education, and connection to a lactation consultant to alleviate J.S.’s fears about buprenorphine/naloxone exposure through breastmilk. By providing supportive care, anticipatory guidance, and ongoing follow‐up, the midwife contributed to J.S.’s continued commitment to provide breastmilk to her newborn postpartum.

Breastfeeding is beneficial to all dyads, but is especially true for those impacted by OUD. Breastfeeding is safe and should be supported for birthing people with OUD, including those who receive MOUD. Despite the known maternal‐infant benefits, breastfeeding continuation rates in this population are lower than the national average. By providing antepartum education and anticipatory guidance about the benefits of providing breastmilk for newborns exposed to opioids, women's health providers can improve the health of families impacted by OUD. Midwives and other maternal and newborn providers are uniquely positioned to support and champion care for dyads who have been exposed to opioids through the provision of stigma‐free, evidence‐based, and whole‐person care.

## CONFLICT OF INTEREST

The authors have no conflicts of interest to disclose.
